# A nanomedicine approach enables co-delivery of cyclosporin A and gefitinib to potentiate the therapeutic efficacy in drug-resistant lung cancer

**DOI:** 10.1038/s41392-018-0019-4

**Published:** 2018-06-22

**Authors:** Weidong Han, Linlin Shi, Lulu Ren, Liqian Zhou, Tongyu Li, Yiting Qiao, Hangxiang Wang

**Affiliations:** 1 Department of Medical Oncology, Sir Run Run Shaw Hospital, School of Medicine, Zhejiang University, 310016 Hangzhou, China; 2 The First Affiliated Hospital, Collaborative Innovation Center for Diagnosis and Treatment of Infectious Diseases, Key Laboratory of Combined Multi-Organ Transplantation, Ministry of Public Health, School of Medicine, Zhejiang University, 310003 Hangzhou, China

## Abstract

Drug resistance, accounting for therapeutic failure in the clinic, remains a major challenge to effectively manage cancer. Cyclosporin A (CsA) can reverse multidrug resistance (MDR), especially resistance to epidermal growth factor receptor tyrosine kinase inhibitors. However, the application of both drugs in cancer therapies is hampered by their poor aqueous solubility and low bioavailability due to oral administration. CsA augments the potency of gefitinib (Gef) in both Gef-sensitive and Gef-resistant cell lines. Here, we show that the simultaneous encapsulation of CsA and Gef within polyethylene glycol-*block*-poly(_D, L_-lactic acid) (PEG-PLA) produced a stable and systemically injectable nanomedicine, which exhibited a sub-50-nm diameter and spherical structures. Impressively, the co-delivery of therapeutics via single nanoparticles (NPs) outperformed the oral administration of the free drug combination at suppressing tumor growth. Furthermore, in vivo results indicated that CsA formulated in NPs sensitized Gef-resistant cells and Gef-resistant tumors to Gef treatment by inactivating the STAT3/Bcl-2 signaling pathway. Collectively, our nanomedicine approach not only provides an alternative administration route for the drugs of choice but also effectively reverses MDR, facilitating the development of effective therapeutic modalities for cancer.

## Cancer: Overcoming drug resistance with nanoparticle delivery

Injection of nanoparticles containing the anticancer drug gefitinib and the immunosuppressant cyclosporin A reverses drug-resistant cancer growth in mice. The development of multidrug resistance is the main reason why many forms of chemotherapy fail. Cyclosporin A, a drug used to prevent immune rejection after organ transplantation, has previously been shown to enhance the potency of gefitinib. Hangxiang Wang and colleagues at Zhejiang University, Hangzhou, China, have successfully combined cyclosporin A and gefitinib, two poorly water-soluble and slowly absorbed drugs, into stable injectable nanoparticles that delay the growth of gefitinib resistant human lung cancer cells as well as the growth of drug resistant tumors in mice. Importantly, this novel co-formulation was more effective than oral co-administration of the two drugs. Further investigation into this drug delivery route could yield much needed alternative treatments for patients with multidrug-resistant cancers.

## Introduction

Despite significant progress in treating cancer, drug resistance remains a major challenge to effectively manage this disastrous disease. Drug resistance also accounts for the therapeutic failure and relapse in over 90% of cancer patients.^1,2^ Cancer drug resistance can be intrinsic or acquired as a response to repeated chemotherapy. Tremendous efforts have been directed toward overcoming this obstacle, including the following strategies: (i) down-regulating the activity of drug efflux pumps with specific inhibitors^3–5^ or RNA interference technology;^6–10^ (ii) developing new therapeutics that are less susceptible to drug resistance;^11,12^ and (iii) combining two or more agents into one treatment scheme, one of which usually rewires specific cell signaling pathways to sensitize the cells and subsequently improves the therapeutic efficacy of the other drugs.^13–16^ The latter anti-resistance approach is particularly attractive because numerous studies have shown that a single magic bullet strategy is a rare commodity in treating cancer. Therefore, drug combinations offer the opportunity to target different pathways in specific populations of cancer cells, thereby maximizing the therapeutic efficacy or overcoming drug resistance mechanisms. However, it is extremely difficult to reconcile the pharmacokinetics, bio-distribution, and cellular uptake of individual drugs that possess distinct physiochemical properties. Therefore, current clinical combinatorial therapy, i.e., simply combining different free drugs, is far from a perfect regimen for cancer patients.

To address these therapeutic challenges, packaging multiple drugs or drug candidates into a single nanocarrier is of interest to achieve synergistic activity. In addition, due to the leaky tumor microvasculature coupled with impaired intratumoral lymphatic drainage, a unique characteristic known as the enhanced permeability and retention (EPR) effect can be leveraged to facilitate the preferential accumulation of nanotherapeutics at tumor site(s), barring drug distribution in normal tissues.^17–21^ Thus, numerous nanostructured vehicles have been explored to efficiently and safely deliver anticancer therapeutics.^22–25^ Particularly, polymeric nanoparticles, as nanocarriers derived from a broad range of amphiphilic copolymers, have shown great potential for drug delivery.^26–29^ Traditionally, these polymeric matrices exhibit excellent biocompatibility and biodegradability, thus avoiding long-term biosafety concerns.^30^ More importantly, multiple structurally diverse hydrophobic drugs can be simultaneously encapsulated within a single platform. Exploiting these nanomedicines has been successful in many instances.^25,31^ However, only a few studies have been conducted to elucidate the effect of delivering a chemosensitizer and a molecular targeted agent (MTA) via a single injectable nanomedicine on therapeutic outcomes in cancer.

Activation of the transcription factor signal transducer and activator of transcription 3 (STAT3) in response to diverse stimuli can promote tumor cell survival and enhance tumor stem cell-like properties.^32–34^ Thus, the targeted disruption of this signal transduction pathway has emerged as a potential therapeutic strategy for cancer. Our previous study has suggested that STAT3 is also associated with chemoresistance against Gef, an epidermal growth factor receptor tyrosine kinase inhibitor (EGFR-TKI), in lung cancer cells.^35^ Strikingly, drug resistance can be partially abolished by the addition of CsA, an Food and Drug Administration (FDA)-approved drug to prevent immune rejection after organ transplantation,^36^ via STAT3 pathway inactivation. We hypothesized and demonstrated that the individual administration of these two drugs could result in reduced drug synergy and, thus, reduce the therapeutic benefits due to uncontrolled pharmacokinetic profiles. However, beyond this preliminary demonstration, we did not study the combinatorial therapeutic effects or attempt the co-encapsulation of these drugs for systemic injection. Therefore, we envisioned that nanomedicine approaches could significantly improve the pharmacokinetics and precisely tailor the intracellular interplay of two drugs, thereby potentiating synergistic efficacy compared with the oral administration or either drug alone.

Herein, by exploiting the intrinsic hydrophobicity of drug payloads, we simultaneously encapsulated a chemosensitizer (i.e., CsA)^37^ and a MTA into polymeric NPs comprising PEG-PLA clinically approved by the FDA.^38–40^ The resulting NPs exhibited significantly improved drug solubility and stability, thereby enabling the systemic injection of both drugs within a single formulation. Moreover, we thoroughly examined the role of CsA in reversing Gef resistance both in vitro and in vivo. In addition to providing an alternative administration route for the drugs of choice, our study also demonstrated that systematically injectable nanomedicines could be utilized as practical and effective therapeutic modalities in oncology.

## Results

### Preparation and characterization of an amphiphilic copolymer-based nanocarrier encapsulating one or two drugs

**Fig. 1:**
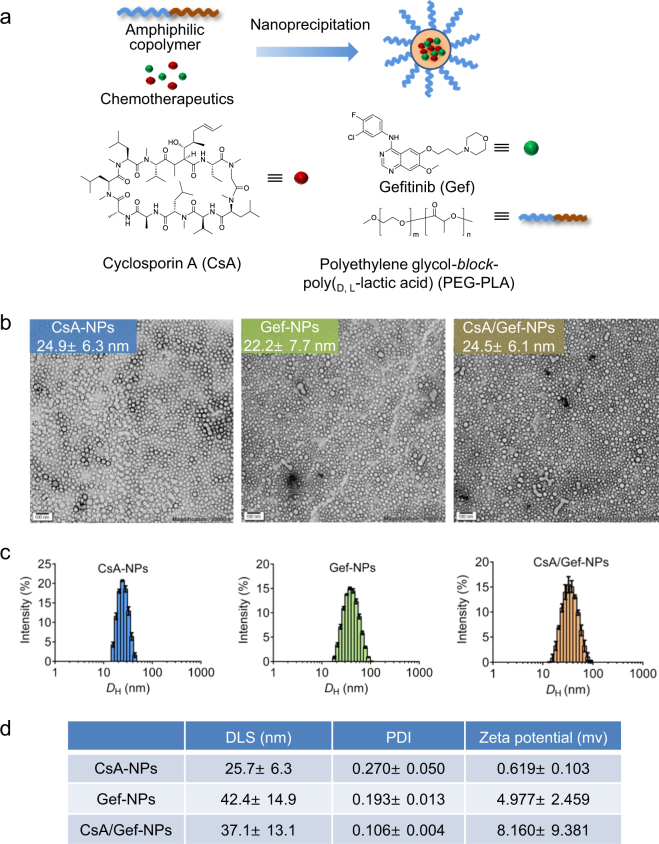
**a** Schematic illustration of the generation of a water-soluble and systemically injectable nanomedicine co-encapsulating two anticancer agents, cyclosporin A (CsA) and Gefitinib (Gef), using the amphiphilic block copolymer polyethylene glycol-block-poly(lactide) (PEG-PLA). The chemical structures of CsA, Gef, and PEG-PLA are also presented. **b** Transmission electron microscopy (TEM) morphology of drug-formulated nanoparticles (NPs) (termed CsA-NPs, Gef-NPs, and CsA/Gef-NPs for formulations including CsA, Gef, and both CsA and Gef, respectively). Scale bars represent 100 nm. **c** Size distribution of NPs as determined by dynamic light scattering (DLS). **d** DLS analysis indicated that these NPs were small in size and had a narrow size distribution. Zeta potentials are also provided

Both chemotherapeutics (CsA and Gef) are highly water insoluble. The solubility of CsA and Gef in deionized (DI) water is less than 0.1 mg/mL.^41,42^ The large void space of the inner core of PEG-PLA NPs was expected to accommodate the integration of the two drugs, thus enabling their systemic injection. To test this rationale, we used a nanoprecipitation procedure to entrap one or two drugs using PEG_5k_-PLA_8k_ matrices **(**Fig. 1a**)**. As expected, blending the PEG_5k_-PLA_8k_ copolymer with CsA, Gef or both CsA and Gef produced transparent solutions rather than precipitates, suggesting the successful encapsulation of chemically dissimilar therapeutics in PEG-PLA NPs. TEM-based morphology studies revealed the formation of spherical nanostructures with a diameter of approximately 25 nm (Fig. 1b). These results were further validated by dynamic light scattering (DLS). DLS analysis showed that the CsA loaded NPs (CsA-NPs), Gef-loaded NPs (Gef-NPs) and CsA- and Gef-coloaded NPs (CsA/Gef-NPs) had average hydrodynamic diameter (*D*_H_) values of 25.7 ± 6.3, 42.4 ± 14.9, and 37.1 ± 13.1 nm, respectively (Fig. 1c, d). More importantly, CsA/Gef-NPs exhibited remarkable colloidal stability in phosphate-buffered saline (PBS) or in the presence of 20% fetal bovine serum (FBS) over long-term incubation (at least 1 week) when stored at room temperature (Fig. S1). Only negligible variations in *D*_H_ were observed for CsA/Gef-NPs as confirmed by DLS analysis.

### In vitro cytotoxicity and chemosensitizing effects of nanomedicine

**Fig. 2:**
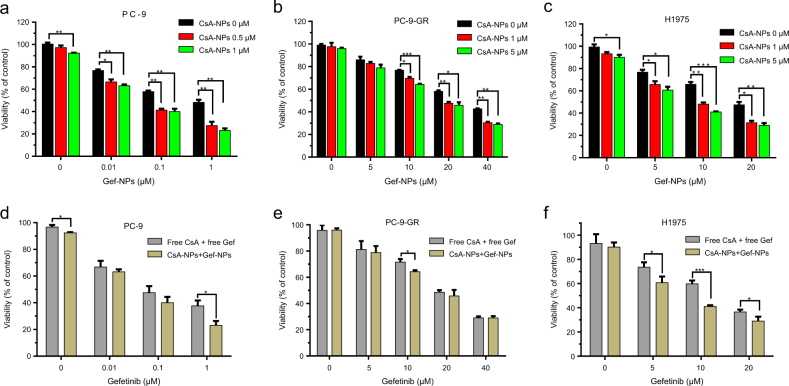
Cytotoxicity of Gef and CsA and their nanoformulations. The viabilities (mean ± SD) of PC-9, PC-9-GR, and H1975 lung cancer cells were determined by the MTT assay in the presence or absence of CsA delivered by NPs. (**a**–**c**) The cells were treated with various formulations in vitro. (**d**–**f**) The in vitro cytotoxicities of the free drug combination and nanodrug combination in successive concentrations were compared in PC-9, PC-9-GR, and H1975 cells. The concentrations of CsA or CsA-NPs included in the nanoformulation or free drugs combination were set to 1 μM, 5 μM, and 5 μM (CsA equivalence) for PC-9, PC-9-GR, and H1975, respectively

Previous studies have indicated that CsA can enhance the potency of MTAs, including Gef.^35,43–45^ We also demonstrated the effect of CsA on sensitizing non-small cell lung cancer (NSCLC) cells to free Gef in Fig. S2. However, we are the first to attempt to verify whether the combination of CsA and Gef could produce a synergistic effect against cancer cells, especially Gef-resistant NSCLC cells, when formulated into NPs and co-delivered. To assess this effect, three NSCLC cell types, including EGFR-TKI-sensitive PC-9, acquired-resistance PC-9 and primary-resistance H1975 cells, were investigated in vitro. After treatment with Gef in the presence or absence of CsA via NPs, cell viability was confirmed by the MTT assay. As shown in Fig. 2a, a remarkable synergistic effect was observed in PC-9 cells even at a low NP concentration of CsA (1 μM CsA equivalent concentration), whereas CsA-NPs alone did not exhibit obvious cytotoxicity. Specifically, the addition of CsA (e.g., 1 μM) to the NP formulation reduced the half-maximal inhibitory concentration (IC_50_) values of Gef from 29.3 to 19.3 μM and from 18.5 to 9.3 μM in Gef acquired resistant PC-9 (PC-9-GR) and primarily Gef resistant H1975 cells, respectively **(**Fig. 2b, c**)**. The cytotoxic effect of the drug-loaded NPs was comparable or even superior to that of the combination of both free drugs in all the tested NSCLC cell types (Fig. 2d–f), indicating that the nanoformulation of the two drugs did not impair the synergistic antitumor effect in vitro.

**Fig. 3:**
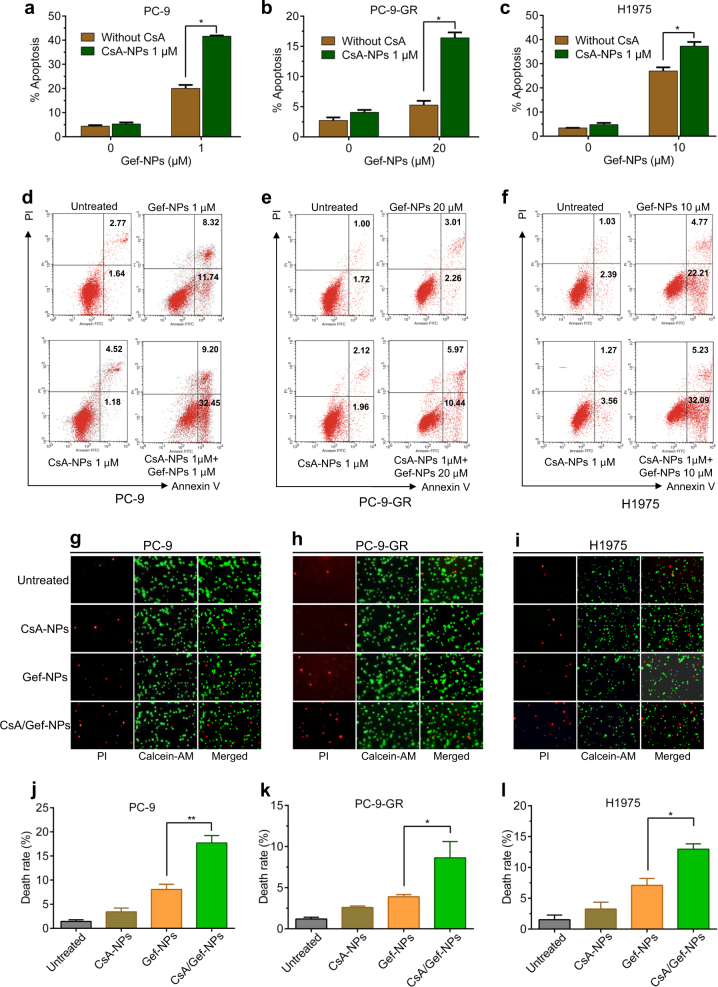
**a**–**f** CsA enhances the cytotoxicity of Gef when co-delivered by NPs to three NSCLC cell lines by promoting apoptosis. Cells were treated with Gef in the absence or presence of CsA via NPs for 48 h before being stained with Annexin V (AV) and propidium iodide (PI), and the apoptotic rates were determined by flow cytometry. The quantitative apoptotic rates are displayed in the upper panel, and the proportions of apoptotic cells are shown in the lower panel. **g**–**i** LIVE/DEAD staining was analyzed by confocal microscopy. PC-9, PC-9-GR, and H1975 NSCLC cells were treated with CsA-NPs (1, 1, 1 μM), Gef-NPs (1, 20, 10 μM), and CsA/Gef-NPs (1 + 1, 1 + 20, 1 + 10 μM). Untreated cells were used as the control. Live cells were stained with calcein-AM, while dead cells were stained with PI. Fluorescence images of the same samples were captured at 490 nm (green) for the Calcein-AM signal and at 545 nm (red) for the PI signal and merged into new images. Scale bars represent 100 μm. **j**–**l** The death rates of PC-9, PC-9-GR and H1975 cells were analyzed. The difference in the death rates of cells treated with CsA-NPs or Gef-NPs alone or in combination was significant

To further investigate whether the enhanced sensitization to Gef by CsA when co-delivered by NPs was a consequence of increased apoptosis, Gef-NPs-treated cells were double stained with propidium iodide (PI)/Annexin V-FITC and quantified by flow cytometry. Treatment with Gef-NPs induced apoptosis, resulting in apoptotic ratios of 20.06, 5.27 and 26.98% in PC-9, PC-9-GR and H1975 cells, respectively. However, the addition of CsA to the NP formulation substantially enhanced the apoptotic ratios to 41.65, 16.41, and 37.32% in the above three cell lines, respectively (Fig. 3a–f**)**. The effect of free CsA on sensitizing cells to free Gef was also confirmed in NSCLC cells (Fig. S3). The synergistic cytotoxic effect of the nanomedicines was further detected by LIVE/DEAD staining. As summarized in Fig. 3g–l, treatment with CsA/Gef-NPs led to 1.8- to 2.3-fold more cell death than treatment with Gef-NPs in all NSCLC cells.

**Fig. 4:**
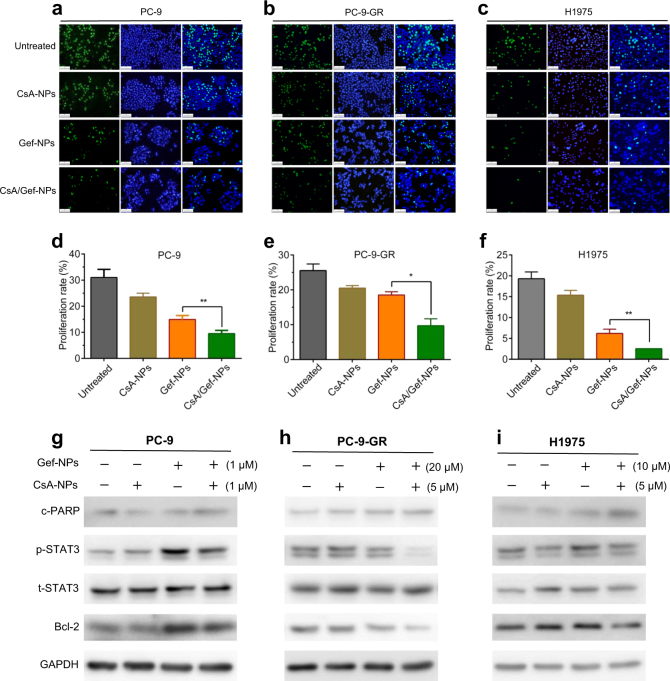
**a**–**f** Inhibitory effect of the nanosystem on cell proliferation. EdU assay results showing the inhibition of cell proliferation by various drugs. **a**–**c** The nuclei of all cells were stained with Hoechst 33342 (blue); only proliferating cells were stained by Alexa Fluor 488 (green). The results were analyzed by fluorescence microscopy (×200), Scale bars represent 100 μm. **d**–**f** The proliferation rates of PC-9, PC-9-GR and H1975 cells treated with CsA-NPs (1, 1, 1 μM), Gef-NPs (1, 20, 10 μM) and CsA/Gef-NPs (1 + 1, 1 + 20, 1 + 10 μM) were quantified and plotted. **g**–**i** CsA sensitized NSCLC cells to Gef when co-delivered by NPs via the inhibition of STAT3/Bcl-2. The expression levels of p-STAT3, t-STAT3, and Bcl-2 were examined by western blotting using PC-9, PC-9-GR, and H1975 cell lysates after treatment with Gef-NPs, CsA-NPs, or CsA/Gef-NPs for 48 h. c-PARP, an apoptosis marker, was also detected by immunoblotting

Additionally, the EdU incorporation assay was applied to examine the effect of the nanomedicine co-encapsulating both drugs on cancer cell proliferation compared with that of the single-drug NP formulations. As presented in Fig. 4a–f, treatment with CsA/Gef-NPs showed a synergistic effect, rendering 1.6-, 1.9-, and 2.4-fold decreases in the proliferation rates compared with Gef-NPs alone in PC-9, PC-9-GR, and H1975 cells, respectively. Taken together, these four independent assays clearly validate the cytotoxic synergy of a chemosensitizer and an MTA regardless of their co-encapsulation in a nanovehicle.

### In vitro mechanism of Gef sensitization by CsA

In accordance with the apoptosis assay, western blot analysis showed that the CsA/Gef-NP treatment increased the level of c-PARP (a marker of apoptosis) compared with that induced by Gef-NP treatment, confirming that CsA promoted apoptosis in combination with Gef when co-encapsulated in NPs (Fig. 4g–i). We further explored the mechanism of Gef sensitization by CsA in this nanoplatform. It has been suggested that STAT3 is extensively correlated with therapeutic resistance to EGFR-TKIs in NSCLC cells and that CsA augments the effect of Gef remarkably by inhibiting STAT3 activation.^35^ Therefore, we evaluated whether the CsA-NP-mediated sensitization to Gef is also dependent on STAT3 inhibition in PC-9, PC-9-GR, and H1975 cells by immunoblotting. Consistent with our previous results, the basal levels of STAT3 in drug-resistant PC-9-GR and H1975 cells were much higher than that in Gef-sensitive PC-9 cells **(**Fig. 4g–i**)**. In PC-9-GR and H1975 cells, Gef-NPs alone had no effect on the p-STAT3 level, while the addition of CsA to the NP formulation resulted in significantly reduced levels of p-STAT3 and its downstream effector Bcl-2. In PC-9 cells, Gef-NP treatment led to the feedback activation of STAT3, which was dramatically suppressed by the addition of CsA to the NP formulation. These results further confirmed that CsA can sensitize cells to Gef when co-delivered by NPs.

### In vivo antitumor activity overcomes drug resistance in a PC-9-GR tumor-bearing mouse model

**Fig. 5:**
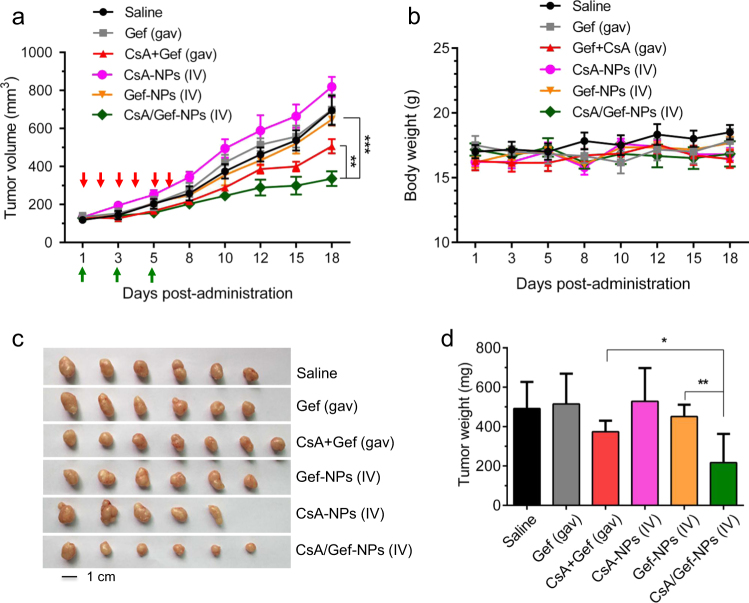
Co-encapsulation of CsA and Gef results in improved therapeutic efficacy in an acquired EGFR-TKI-resistant PC-9-GR tumor-bearing mouse model. **a** The tumor volume (mm^3^) change is presented as a function of time after drug treatment. When the tumor volume reached approximately 120 mm^3^, NP solutions were IV injected three times (indicated by green arrows). Free CsA and/or free Gef were orally administered (shown in red arrows). Saline was intravenously injected as a control. **b** Body weight gain/loss profiles of mice upon treatment. **c** Image showing tumors excised from the mice. **d** The tumor tissue weight on day 18, the endpoint of the study

To further evaluate the therapeutic efficacy of NPs containing both CsA and Gef, we established an NSCLC xenograft-bearing BALB/c nude mouse model by implanting EGFR-TKI-resistant PC-9-GR cells into immunodeficient mice. When the tumor volume reached approximately 120 mm^3^, the mice were administered drugs via the tail vein. The therapeutic results are shown in Fig. 5a. Free Gef or Gef-NPs alone did not observably inhibit the growth of PC-9-GR tumor xenografts that had acquired EGFR-TKI resistance. Similarly, CsA-NPs alone did not effectively suppress tumor growth. As expected, the combination of free CsA and Gef administered by gavage delayed tumor growth compared with free Gef alone. More strikingly, the intravenous (IV)-administered nanomedicine (i.e., CsA/Gef-NPs) suppressed tumor growth more effectively than the conventional regimen of combined oral administration (Fig. 5a**)**. As indicated in Fig. 5c, d, the tumor volume of mice treated with CsA/Gef-NPs was dramatically smaller than those of mice treated with the combination of free drugs (1.7-fold decrease) and mice treated with saline (2.6-fold decrease) on day 18 post-administration. It should be noted that the total dosage of the CsA plus Gef administered orally was twofold greater than that of the IV injected CsA/Gef-NPs. Furthermore, no significant body weight loss was observed in mice receiving the CsA/Gef-NPs, indicating that the injected NP formulation had low systemic toxicity **(**Fig. 5b**)**.

### In vivo inhibition of STAT3/Bcl-2 in mice by CsA-NP treatment

**Fig. 6:**
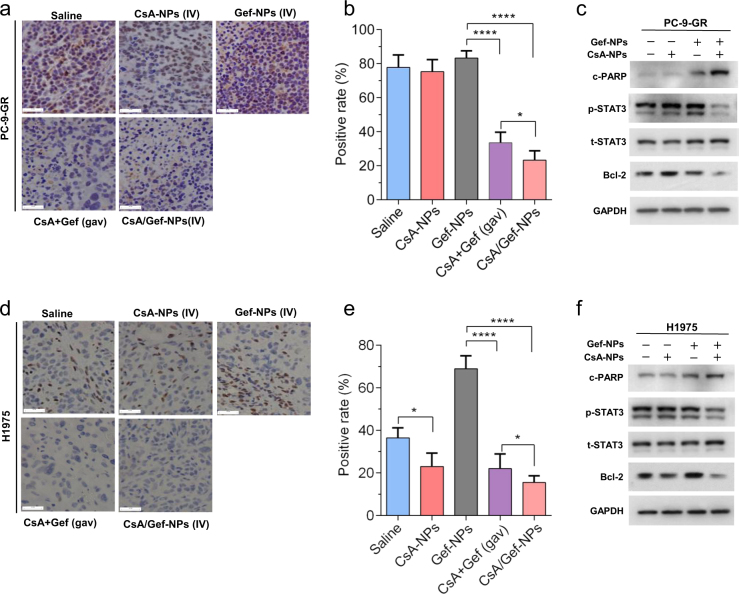
CsA inhibits Gef-induced STAT3 activation when co-delivered by NPs in two NSCLC xenograft models. Scale bars represent 50 μm. **a**–**c** Immunohistochemical (IHC) staining for p-STAT3 in paraffin-embedded tissue sections, quantitative IHC results, and western blot results for p-STAT3/Bcl-2 in PC-9-GR xenograft-bearing mice. **d**–**f** The same assays were conducted for H1975 xenografts. The magnification of the IHC images is 400×. **c**, **f** Tumor tissue levels of c-PARP, an apoptosis marker, in both PC-9-GR and H1975 xenograft tumors were also detected by immunoblotting

Finally, we investigated the effects of CsA-NPs on the STAT3 signaling pathway in nude mice bearing PC-9-GR and H1975 xenograft tumors. The tumor tissues of mice administrated with the drug formulations were collected on day 18 and subjected to histological analysis. In accordance with our previous report^35^ and the in vitro analyses described above, the p-STAT3 levels in mice treated with the combination of free drugs were dramatically lower than those in mice treated with Gef-NPs alone. Moreover, the co-delivery of CsA and Gef in NPs further decreased the p-STAT3 levels compared with the oral administration of the drug combination by 1.52- and 1.44-fold in H1975 and PC-9-GR xenograft tumors, respectively (Fig. 6a, b, d, e). To validate the enhanced effect, we analyzed proteins extracted from the excised tumors by western blotting. As expected, the levels of both p-STAT3 and downstream Bcl-2 were significantly reduced in mouse tumors after treatment with CsA/Gef-NPs. On the other hand, c-PARP was evidently activated in two distinct tumor tissues after treatment with CsA/Gef-NPs, indicating that the inhibition of STAT3 by CsA augmented the Gef-induced apoptosis of tumor cells (Fig. 6c, f), a finding that is consistent with the observed potent suppression of tumor growth.

## Discussion

MDR is one of the largest concerns for cancer therapies. To this end, we have described the co-formulation of the MDR-reversing agent CsA and MTA Gef into a single PEG-PLA NP platform. Characterization of the PEGylated nanoplatform showed that NPs prepared by this method exhibited sub-50-nm diameters (Fig. 1), enabling them to deeply penetrate solid tumors via the EPR effect. Additionally, cloaking the surface with PEG chains not only provides aqueous solubility but also confers the NPs with a “stealth” property, thereby extending the duration of NP circulation in the blood.

The results from a series of in vitro studies demonstrated that the addition of CsA to the NP formulation increased the potency of Gef in non-Gef-resistant cells as well as in primarily and secondarily Gef-resistant cells. The apoptosis, western blotting and EdU assays further showed that the sensitization to Gef by CsA when co-delivered by NPs was a consequence of increased apoptosis and impaired proliferation of NSCLCs. Compared with the free drug combination administered by gavage in vivo, the IV-administered drug combination of CsA and Gef in nanoform significantly decreased tumor growth (Fig. 5c). Furthermore, no significant body weight loss was observed in mice receiving the nanoformulated drug combination, indicating that the injected NP formulation had low systemic toxicity (Fig. 5b).

**Fig. 7:**
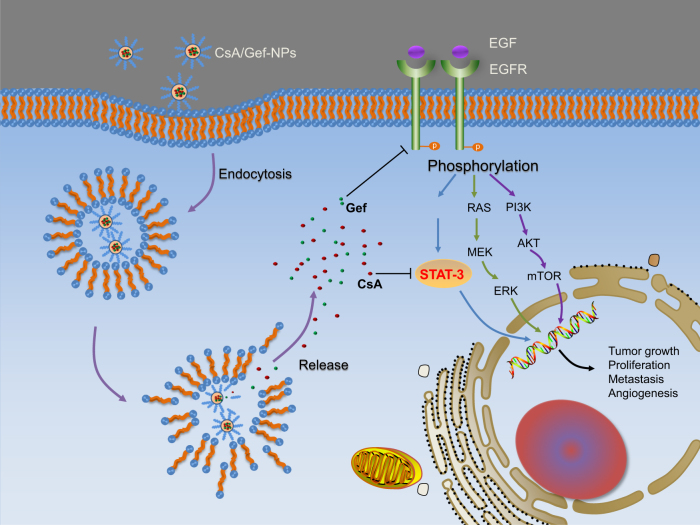
General mechanism explaining how CsA/Gef-NPs overcome MDR and suppress cancer

The mechanisms accounting for the high potency of CsA in sensitizing Gef in the nanoformulation were also investigated. As indicated in Fig. 4g–i, Gef-NPs alone had no effect on the p-STAT3 level in PC-9-GR and H1975 cells, while the addition of CsA to the NP formulation resulted in significantly reduced levels of p-STAT3 and its downstream effector Bcl-2. The same sensitization effect was also witnessed in vivo (Fig. 6). Those mechanisms are summarized in Fig. 7. As a proproliferative, proinvasive and antiapoptotic protein on the cell membrane, the dimerization and autophosphorylation of EGFR stimulate its intrinsic intracellular protein tyrosine kinase activity and initiates downstream pleiotropic oncogenic signaling cascades, including the Ras/MEK/ERK,^46,47^ PI3K/Akt/mTOR,^25,26,48,49^ and STAT3 pathways.^50,51^ Gef inhibits EGFR tyrosine kinase and leads to the disruption of downstream signaling transduction, thus inhibiting the growth and invasion of tumor cells. Exposure to Gef results in the feedback activation of STAT3, which is known to promote tumor cell survival under conditions of stress. However, CsA co-delivered with Gef by NPs is released from the CsA/Gef-NPs and subsequently reverses the activation of STAT3 to effectively augment Gef-induced apoptosis.

In conclusion, our NP-mediated drug codelivery approach successfully overcame MTA resistance by rewiring specific signaling pathways and outperformed the combination of free drugs, enlightening our researchers on overcoming MDR and exploring alternative drug administration routes.

## Materials and methods

### Materials

Polyethylene glycol-*block*-poly(_D, L_-lactic acid) (PEG_5k_-*b*-PLA_8k_) was purchased from Advanced Polymer Materials, Inc. (Montreal, Canada). Gef (184475-35-2) and CsA (59865-13-3) were purchased from J&K Scientific (Beijing, China). All other compounds and solvents were purchased from J&K Chemical (Shanghai, China) and utilized without further purification or dilution.

### Preparation and characterization of NPs

Polymeric NPs were prepared *via* the nanoprecipitation method^52–54^. To fabricate CsA/Gef-NPs, 1 mg of CsA and 40 mg of PEG-PLA were dissolved in 2 ml of acetone, while 1 mg of Gef was dissolved in 0.1 ml of dimethyl sulfoxide (DMSO). Next, the solution was mixed and added dropwise into 10 ml of DI water while stirring. After stirring for 30 min, the solution was evaporated in a rotary evaporator at reduced pressure at 30 °C for ~20 min to remove acetone, yielding a final polymer concentration of 0.1 mg/mL. The transparent solution containing NPs was concentrated with an Amicon Ultra-4 centrifugal filter (MWCO 10 kDa; UFC800324, Millipore, Germany) and washed with DI water. The concentration of copolymer was determined by high-performance liquid chromatography. CsA-NPs and Gef-NPs were prepared by the same method. The *D*_*H*_, polydispersity index (PDI), and ζ potential of the drug-loaded micelles were measured using a Malvern Nano-ZS 90 laser particle size analyzer at 25 °C. The morphological characteristics of the NPs were observed by transmission electron microscopy (TEM; H-6009IV, Hitachi, Japan). Samples for TEM were stained with 1% uranyl acetate.

### Cell lines and cell culture

The NSCLC cell lines PC-9, which possesses an EGFR-activating mutation exon 19 deletion and H1975, which harbors the EGFR-activating mutation L858R and the resistant mutation T790M, were purchased from the cell bank of the Chinese Academy of Science (Shanghai, China). PC-9-GR cells were obtained according to our previous report.^35^ Cells were maintained in Dulbecco’s Modified Eagle’s Medium (DMEM; Gibco, Carlsbad, CA, USA) at 37 °C in a 90% humidified atmosphere with 5% CO_2_. All media were supplemented with 10% FBS, penicillin (100 units/mL), and streptomycin (100 mg/mL). Antibiotics, FBS, 0.25% (w/v) trypsin, and the 0.03% (w/v) EDTA solution were purchased from HyClone.

### Cell viability and proliferation assays

#### MTT assay

Cells were seeded in 96-well plates (5000 cells and 100 μL of media per well) and incubated at 37 °C overnight. Subsequently, the adherent cells were treated with different concentrations of free Gef, free CsA, CsA-NPs, Gef-NPs, or CsA/Gef-NPs. Cells treated with medium that contained the same amount of DMSO as the drug treatment group were included as controls. After 48 h of incubation, 30 μL of 3-[4,5-dimethylthiazol-2-yl]-3,5-diphenyl tetrazolium bromide (MTT) solution (5 mg/mL in PBS) was added to each well. After 4 h of incubation at 37 °C, the MTT solution was removed from the wells, followed by the addition of 100 μL of DMSO per well. The absorbance of individual wells was then measured at 492 nm using a microplate reader (Multiskan FC, Thermo Scientific) after 2 h of incubation. All experiments were conducted in triplicate. The IC_50_ was calculated using GraphPad Prism 6.0. Untreated cells served as controls, and their viability was defined as 100%. Cell viability was calculated using the following formula: cell viability = (A sample/A control) × 100%, where A represents the absorbance at 492 nm. The results were from three repeated independent experiments.

### LIVE/DEAD staining assay

For the LIVE/DEAD staining assay, cells were seeded in 6-well plates at a cell density of 2 × 10^5^/well. After treatment with different concentrations of NPs for 48 h, the cells in each well were collected and re-suspended in 200 μL of PBS. Thereafter, the cells were stained with 1 μL of staining solution containing calcein-AM (2 μM) and 1 μL of PI (4 μM), which stains apoptotic/dead cells. After 30 min of incubation in the dark at 37 °C, the cells were imaged using a fluorescence microscope. The percentage of red (dead) cells among all cells (green + red) represents the cell death ratio.

### EdU assay

Cell proliferation and DNA synthesis were determined using a Click-iT® EdU Alexa Fluor® 488 Assay Kit (Invitrogen) according to the manufacturer’s protocol. Briefly, cells (2 × 10^4^ cells/well) were seeded in 48-well plates and cultured overnight before being exposed to different concentrations of CsA-NPs, Gef-NPs or CsA/Gef-NPs for 48 h. Cells treated with DMEM alone were used as the control. Next, 100 μL of EdU (10 μM) was added to each well, followed by 4 h of incubation at 37 °C. After washing with PBS three times, the cells were fixed with 4% paraformaldehyde in PBS for 30 min and permeabilized with 0.5% Triton X-100 in PBS for 30 min at room temperature. Thereafter, 100 μL of Alexa Fluor 488 staining solution was added to each well, followed by incubation for 30 min in the dark at room temperature. After the solution was removed, 100 μL of Hoechst 33342 (5 μg/mL) nuclear staining solution was added to each well, followed by incubation for 10 min. Finally, the cells were visualized by fluorescence microscopy. The ratio of EdU-positive cells (green) to all Hoechst-positive cells (blue) represents the proliferation ratio.

### Apoptosis analysis using flow cytometry with Annexin V-FITC

To analyze apoptosis, cells were seeded in 6-well plates at a density of 2.0 × 10^5^ cells/well and allowed to grow overnight. Next, the cells were treated with different drugs at different concentrations for 48 h at 37 ℃. Untreated cells were used as the control. After incubation, the cells were harvested and washed twice with cold PBS. Next, 1 × 10^5^ cells were dispersed in 100 μL of 1 × Annexin V binding buffer. Subsequently, 5 μL of Annexin V-FITC and 5 μL of PI were added, and the cells were incubated at room temperature in the dark for 15 min. Finally, 400 μL of 1 × Annexin V binding buffer was added under gentle mixing, and the samples were analyzed by flow cytometry (BD Biosciences, San Jose, CA).

### Western blot analysis

Cells were cultured at a density of 2.0 × 10^5^ cells/well in a 6-well plate and allowed to grow overnight. Next, following treatment with free Gef, free CsA, CsA-NPs, Gef-NPs, or CsA/Gef-NPs, the cells were harvested and lysed in RIPA lysis buffer supplemented with complete protease inhibitor cocktail tablets on ice. Protein was also extracted from the tumor tissues of mice in the in vivo study. Protein concentrations were determined by the bicinchoninic acid protein assay. An equivalent amount of protein was taken from each sample, separated by sodium dodecyl sulfate–polyacrylamide gel electrophoresis, transferred onto polyvinylidene difluoride membranes, and then incubated with antibodies (Cell Signaling Technology) against cleaved poly(ADP-ribose) polymerase (c-PARP), phosphorylated STAT3 (p-STAT3), total STAT3 (t-STAT3), B cell lymphoma-2 (Bcl-2), and glyceraldehyde-3-phosphate dehydrogenase (GAPDH). The blots were developed with horseradish peroxidase-conjugated secondary anti-rabbit IgG or anti-mouse IgG antibody (Cell Signaling Technology) and visualized with a chemiluminescent substrate on X-ray films (Kodak). Western blotting of each protein was performed at least three times.

### In vivo antitumor activity

Four- to five-week-old male BALB/c nude mice were purchased from the Shanghai Experimental Animal Center of the Chinese Academy of Science and used for NSCLC implantation. In total, 100 μL of the PC-9-GR and H1975 cell suspensions (5 × 10^6^ cells) was subcutaneously injected into the right flank of each mouse. When the tumor volume reached approximately 120 mm^3^, the mice were randomized into treatment groups. Mice bearing H1975 xenografts were assigned into five groups for histological study, whereas mice bearing PC-9-GR xenografts were randomized into 11 groups (6 groups for tumor growth evaluation and 5 groups for histological study). Each group contained five to seven mice. NP solutions containing the combination of CsA and Gef (at a dose of 10 mg/kg for each drug) were IV injected every other day three times, while the combination of the two free drugs was administered by gavage (gav) (at a dose of 20 mg/kg for each drug) successively for 6 days. CsA-NPs (IV, three times, 10 mg/kg CsA equivalence), Gef-NPs (IV, three times, 10 mg/kg Gef equivalence), Gef (gav, six times, 20 mg/kg), and saline were included as references. The tumor volume (*V*) was calculated using the following formula: *V* = (*L* × *W*^2^) × 0.5, where *L* represents the length, and *W* represents the width. The weight of each mouse was measured for the evaluation of systemic toxicity. At the end of the study, the mice were sacrificed by CO_2_ inhalation, and the tumor tissues were fixed in formalin and embedded in paraffin. All the animal protocols were conducted in compliance with the National Institute’s Guide for the Care and Use of Laboratory Animals.

### Histopathological analysis of tumor tissues

Immunohistochemical (IHC) staining using primary antisera and avidin-biotin-peroxidase complex methods were performed using formalin-fixed tumor sections. p-STAT3 expression was monitored using antibodies and visualized by light microscopy. Six fields of view for each sample (magnification, 400×) were randomly selected and analyzed by three pathologists.

### Statistical analysis

All the data are presented as means ± SD and were analyzed using SPSS 17.0 software. The significance of differences was assessed using one-way ANOVA combined with Student’s *t*-test (*p < 0.05; **p < 0.01; ***p < 0.001).

## Electronic supplementary material

supplementary information

read me-how to open supplementary information
