# Supplementary material for: A nanomedicine approach enables co-delivery of cyclosporin A and gefitinib to potentiate the therapeutic efficacy in drug-resistant lung cancer
Source: Signal Transduct Target Ther. 2018 Jun 22;3:16. doi: 10.1038/s41392-018-0019-4 (PMC6013461; doi:10.1038/s41392-018-0019-4)
Supplement: Supplementary file 1 — supplementary information [file 41392_2018_19_MOESM1_ESM.doc]

Supplementary Information

Nanomedicine Approach Enables Co-Delivery of Cyclosporin A and Gefitinib for Potentiating Therapeutic Efficacy in Drug-Resistant Lung Cancer

*Weidong Han,^a^ Linlin Shi,^a^ Lulu Ren,^a^ Liqian Zhou,^b^ Tongyu Li,^b^ Yiting Qiao,^b^ and Hangxiang Wang,^b^,**

*^a^ Department of Medical Oncology; Sir Run Run Shaw Hospital; School of Medicine, Zhejiang University; Hangzhou, 310003, P.R. China.*

*^b^ The First Affiliated Hospital; Collaborative Innovation Center for Diagnosis and Treatment of Infectious Diseases; Key Laboratory of Combined Multi-Organ Transplantation, Ministry of Public Health, School of Medicine, Zhejiang University, Hangzhou, 310003, P.R. China.*

**Address correspondence to Hangxiang Wang (wanghx@zju.edu.cn)*

*
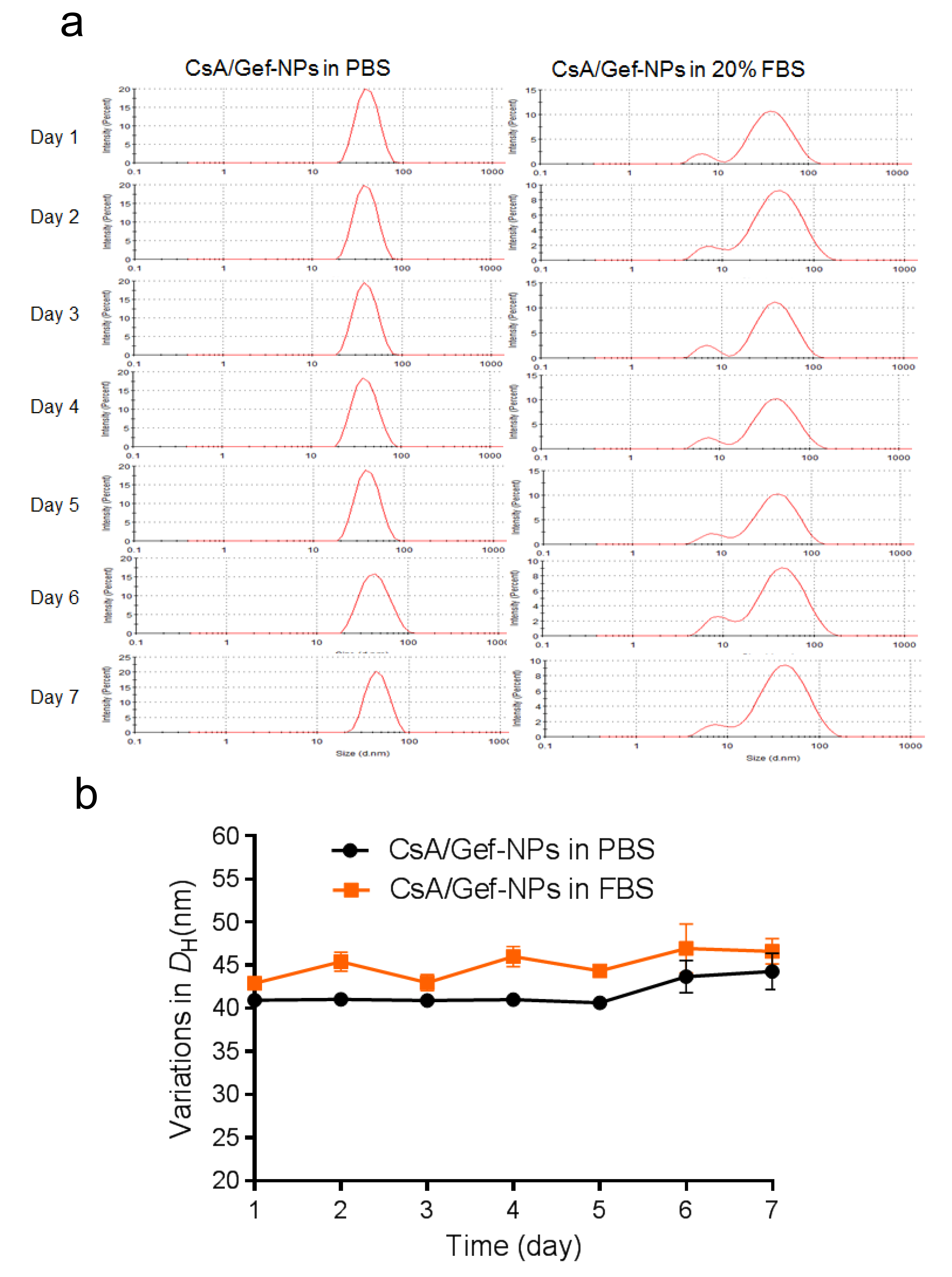
*

**Figure S1.** The stability of CsA/Gef-NPs under different conditions examined by dynamic light scattering (DLS) analysis. (a) Hydrodynamic diameters (*D*_H_) of CsA/Gef-NPs in phosphate-buffered saline (PBS) or in the presence of 20% fetal bovine serum (FBS) were measured every day by DLS. (b) The *D*_H_ variation for CsA/Gef-NPs is presented over one week incubation.


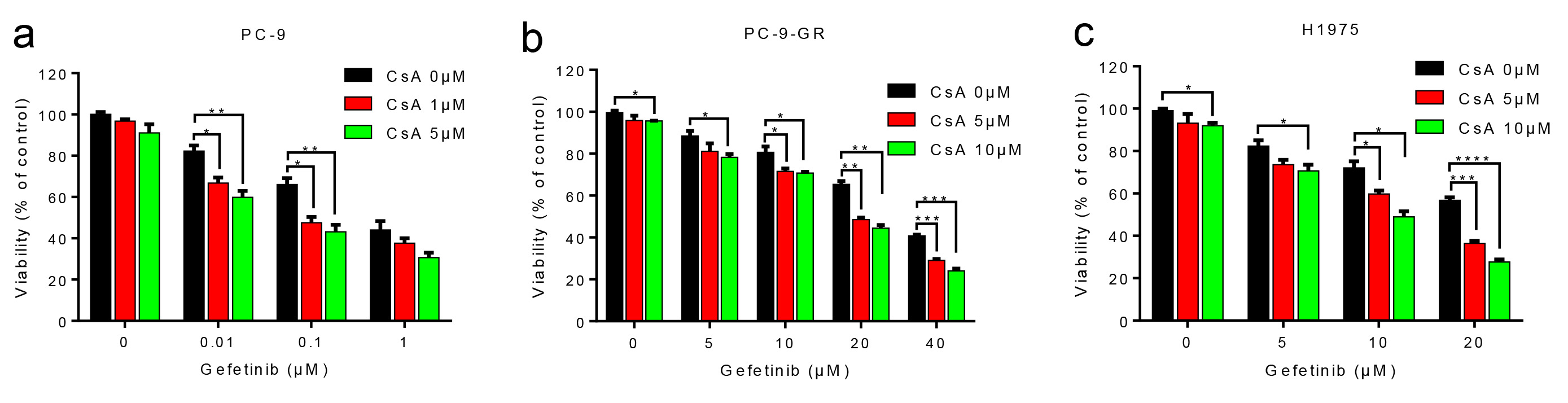
**Figure S2.** Free CsA augments the anti-cancer effect of gefitinib in NSCLC cells. The viability (mean ± SD) of (a) PC-9, (b) PC-9-GR and (c) H1975 lung cancer cells was determined by MTT assay in the presence or absence of CsA.


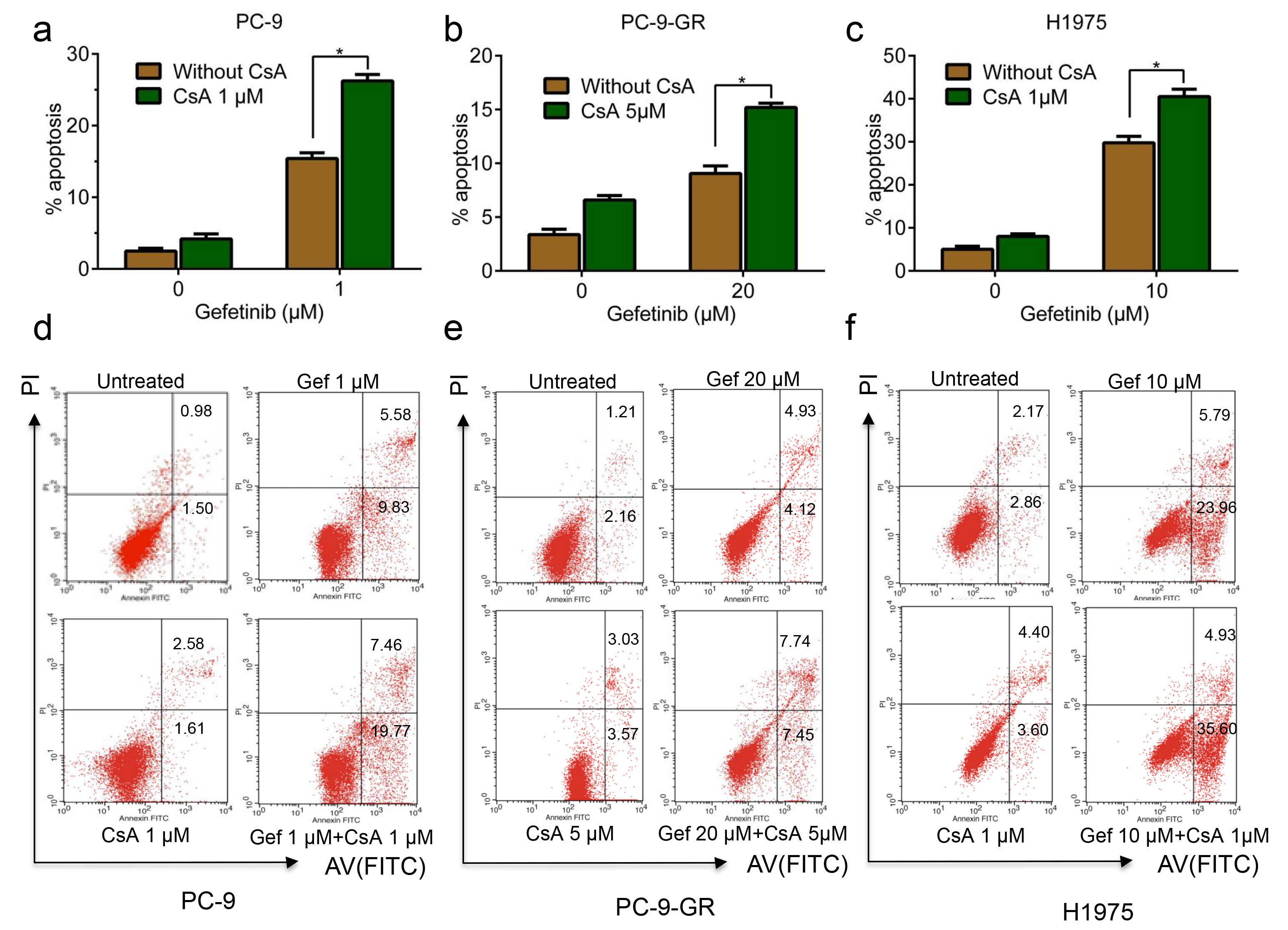


**Figure S3.** Free CsA sensitizes free gefitinib in NSCLC cells through promoting apoptosis. Cells were treated with Gef in the absence or presence of CsA for 48 h before being stained with Annexin V (AV) and propidium iodide (PI), and the apoptotic rates were determined by flow cytometry. (a-c) The quantitative apoptotic rates of PC-9, PC-9-GR and H1975 lung cancer cells are displayed. (d-f) The proportions of apoptotic cells of PC-9, PC-9-GR and H1975 lung cancer cells are shown in the lower pane.
