# Supplementary material for: A nanomedicine approach enables co-delivery of cyclosporin A and gefitinib to potentiate the therapeutic efficacy in drug-resistant lung cancer
Source: Signal Transduct Target Ther. 2018 Jun 22;3:16. doi: 10.1038/s41392-018-0019-4 (PMC6013461; doi:10.1038/s41392-018-0019-4)
Supplement: Supplementary file 2 — read me-how to open supplementary information [file 41392_2018_19_MOESM2_ESM.doc]

Supplementary information is all presented in a doc file and can be opened by Microsoft Office.
